# Effect of an Education Programme for South Asians with Asthma and Their Clinicians: A Cluster Randomised Controlled Trial (OEDIPUS)

**DOI:** 10.1371/journal.pone.0158783

**Published:** 2016-12-28

**Authors:** Chris Griffiths, Stephen Bremner, Kamrul Islam, Ratna Sohanpal, Debi-Lee Vidal, Carolyn Dawson, Gillian Foster, Jean Ramsay, Gene Feder, Stephanie Taylor, Neil Barnes, Aklak Choudhury, Geoff Packe, Elizabeth Bayliss, Duncan Trathen, Philip Moss, Viv Cook, Anna Eleri Livingstone, Sandra Eldridge

**Affiliations:** 1 Asthma UK Centre for Applied Research, Centre for Primary Care and Public Health, Barts and The London School of Medicine and Dentistry, Queen Mary University of London, United Kingdom; 2 Centre for Academic Primary Care, School of Social and Community Medicine, University of Bristol, Canynge Hall, Bristol, United Kingdom; 3 William Harvey Research Institute, Barts and The London School of Medicine and Dentistry, Queen Mary University of London, United Kingdom; 4 Newham University Hospital, Barts and the London NHS Trust, Plaistow, London, United Kingdom; 5 Social Action for Health, The Brady Centre, London; 6 Newham Transitional Practice, Newham, London, United Kingdom; 7 St George’s University Hospitals NHS Foundation Trust, London, United Kingdom; UNM Cancer Center, UNITED STATES

## Abstract

**Background:**

People with asthma from ethnic minority groups experience significant morbidity. Culturally-specific interventions to reduce asthma morbidity are rare. We tested the hypothesis that a culturally-specific education programme, adapted from promising theory-based interventions developed in the USA, would reduce unscheduled care for South Asians with asthma in the UK.

**Methods:**

A cluster randomised controlled trial, set in two east London boroughs. 105 of 107 eligible general practices were randomised to usual care or the education programme. Participants were south Asians with asthma aged 3 years and older with recent unscheduled care. The programme had two components: the Physician Asthma Care Education (PACE) programme and the Chronic Disease Self Management Programme (CDSMP), targeted at clinicians and patients with asthma respectively. Both were culturally adapted for south Asians with asthma. Specialist nurses, and primary care teams from intervention practices were trained using the PACE programme. South Asian participants attended an outpatient appointment; those registered with intervention practices received self-management training from PACE-trained specialist nurses, a follow-up appointment with PACE-trained primary care practices, and an invitation to attend the CDSMP. Patients from control practices received usual care. Primary outcome was unscheduled care.

**Findings:**

375 south Asians with asthma from 84 general practices took part, 183 registered with intervention practices and 192 with control practices. Primary outcome data were available for 358/375 (95.5%) of participants. The intervention had no effect on time to first unscheduled attendance for asthma (Adjusted Hazard Ratio AHR = 1.19 95% CI 0.92 to 1.53). Time to first review in primary care was reduced (AHR = 2.22, (1.67 to 2.95). Asthma-related quality of life and self-efficacy were improved at 3 months (adjusted mean difference -2.56, (-3.89 to -1.24); 0.44, (0.05 to 0.82) respectively.

**Conclusions:**

A multi-component education programme adapted for south Asians with asthma did not reduce unscheduled care but did improve follow-up in primary care, self-efficacy and quality of life. More effective interventions are needed for south Asians with asthma.

## Introduction

Compared with majority populations, minority ethnic groups often have poorer outcomes for long term conditions.[1] Finding effective interventions to address these health inequalities is an important challenge. In the UK, people of South Asian origin with asthma experience excess morbidity, with hospitalisation rates up to three times those of majority white populations.[2–4] Causes of excess morbidity include poor access to healthcare and cultural attitudes to, and beliefs about, illness. South Asians with asthma report difficulties registering with a general practitioner and accessing both routine appointments and emergency care.[5] Follow up in primary care is poor after hospitalisation, and aspects of management and education may be suboptimal.[6, 7] Stigma and unhelpful illness beliefs also contribute: compared to whites, south Asian parents of children with asthma are less likely to reveal that their child has asthma, are less likely to give preventive medication, and more likely to hold the view that medication is harmful and addictive.[8]

Most educational interventions to improve asthma care are directed at majority populations, potentially widening health inequalities. Of the few culturally specific interventions for asthma tested in randomised trials,[9–18] only one (a negative study set in the UK) has targeted south Asians.[15] Most have been set in the USA and directed at African American or Hispanic populations, with inconsistent improvements in quality of life[12, 17, 19] and health care use.[11, 12, 16, 18]

Culturally-specific interventions can be developed *de novo*, or adapted from existing interventions that are effective in majority populations. They can target providers or patients. Two theoretically-based educational interventions that could improve asthma outcomes for south Asians are the Professional Asthma Care Education programme (PACE)[20] and the Chronic Disease Self Management Programme (CDSMP).[21] Both were developed and tested in the USA. PACE is directed at clinicians and comprises a multi-faceted seminar based on self-regulation theory.[20] Goals of the PACE programme are to improve the effectiveness of asthma consultations by improving communication, promoting shared decision-making, and delivering positive messages to address unhelpful beliefs about asthma. PACE reduced emergency care for asthma in the USA[20, 22, 23], has long term impact,[24] is recommended by the American Asthma Association, and is being adapted for use with American ethnic minority populations.[25]. The CDSMP is directed at patients, is based on socio-cognitive theory,[21] is delivered largely by lay trainers, and may have long term impact on health care use for people with long term conditions,[21, 26] There is considerable interest in the potential impact of PACE and the CDSMP outside the USA. PACE has been used (with less impact) in Australia,[27] and is being evaluated for use by Australian pharmacists.[28] The CDSMP is the basis of the UK’s Expert Patient Programme.[29, 30] Lay-led interventions are modestly effective.[31, 32] We showed that an adapted version of the CDSMP, delivered by trained local south Asian lay leaders, improved self-efficacy in south Asians in the UK with a range of chronic illness, including asthma.[33]

PACE and the CDSMP potentially complement each other, by targeting clinicians and patients respectively. We hypothesised that these programmes, when culturally-adapted for South Asians and delivered to primary and secondary care clinicians (PACE) and to south Asians with poorly controlled asthma (CDSMP), could improve asthma outcomes and, in particular, reduce unscheduled care. We tested this in a cluster randomised trial in a socioeconomically deprived, multi-ethnic area in east London. We chose a cluster design, randomising general practices, because a major component of the intervention was delivered at general practices.

## Methods

The study was approved by the east London NHS research ethics committee (ref Q0603/14) and is registered at ClinicalTrials.gov: NCT00214669. Fully informed written consent was obtained from all participants.

### Interventions

**Fig 1:**
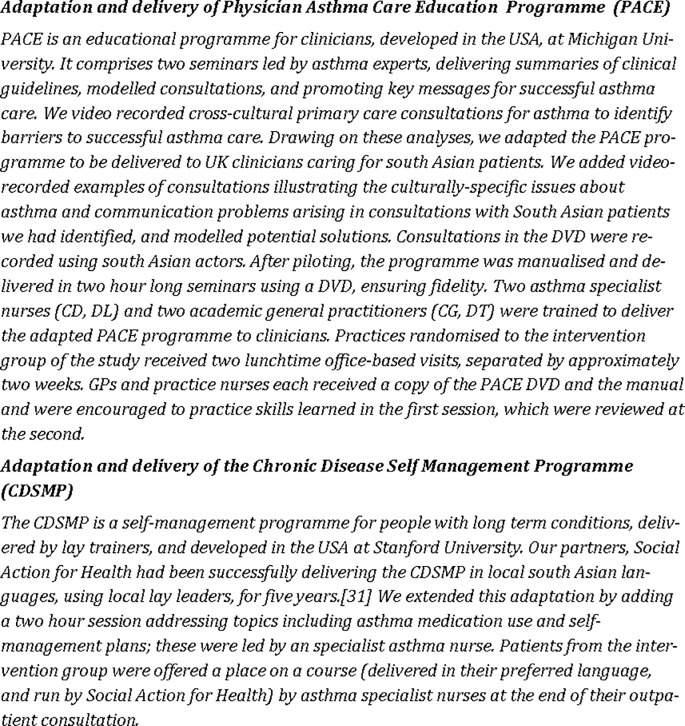
Adaptation of educational programmes

We adapted the Physician Asthma Care Education (PACE) programme, and the Chronic Disease Self Management Program (CDSMP) for use in south Asians with asthma (Fig 1).

### Practice recruitment

We approached all general practices in the east London boroughs of Tower Hamlets and Newham. 105 of 107 agreed to take part.

### Randomisation

We randomised practices to usual care or the education programme, stratifying practices by borough, and using a minimisation programme that maintained allocation concealment. With this programme there was an 80% probability that the next practice to be allocated would be to the group that minimised the imbalance across the minimisation factors. The goal of randomisation by cluster was to deliver two groups of general practices, the characteristics of which were similar in terms of clinicians and patients. Minimisation variables were: the number of GP partners, the practice’s hospital admission rate for asthma, the percentage of the practice list made up of south Asians, and the numbers of south Asians registered with the practice. Each was divided into tertiles. The design meant that clinicians and patients could not be blinded to allocation.

### Patient recruitment

Two researchers fluent in south Asian languages (RS, KI) and blinded to practice allocation invited all south Asian adults and children (aged over 3 years) registered with study practices and attending hospital and out of hours services with an asthma exacerbation to take part. Adults agreeing to take part provided written informed consent; for minors, parents / guardians provided written informed consent on their behalf. Participants were invited to attend a hospital outpatient appointment.

### Patients from intervention practices

At this out-patient appointment, patients from intervention practices saw a PACE-trained asthma specialist nurse. The nurse provided a consultation focusing on delivery of key messages about asthma and agreement of short and long term goals for asthma. Patients discussed, and where capable of using one, received a written self management plan for asthma, translated into their mother tongue, if required (Asthma UK). To improve follow up in primary care, asthma specialist nurses telephoned the patient’s general practice to make them an appointment with their PACE-trained GP or practice nurse. Specialist nurses also invited patients to attend a CDSMP. If they agreed, their names were passed to a co-ordinator, who offered them a CDSMP at a convenient time and location.

### Patients from control practices

At out-patient appointments, patients from control practices saw an asthma specialist nurse who delivered a standardised structured consultation comprising instruction on inhaler use, reinforcement of adherence, and answering of questions posed. No self management plans were provided and no follow up appointments were scheduled.

### Outcomes and assessment

#### Primary outcomes

Co-primary outcomes were: unscheduled care as 1) time to first unscheduled contact with an asthma exacerbation, and 2) proportion of participants without unscheduled care. Unscheduled care was defined as emergency admission, A&E attendance, GP out-of-hours or walk-in centre asthma consultation, or GP consultation comprising presentation with acute asthma-related symptoms, with increase in asthma medication or antibiotic prescription.

#### Secondary outcomes

Secondary outcomes were:

1. time to first asthma review in primary care
2. asthma-specific and generic health related quality of life, using AQ20,[34] North of England,[35] and EQ5D scales[36]
3. Prescribing assessed from patient records and interviews.

### Data collection

#### Medical records

We visited participating practices one year after patient recruitment and copied GP medical records. Records were anonymised and data extracted by blinded researchers, with validation of 10% random sample by another blinded researcher. We extracted data for the year before, and year after, intervention.

#### Interviews

Two researchers, blinded to randomisation status, interviewed participants in person at baseline and by telephone at three and 12 months after recruitment to gather data on quality of life and asthma symptoms. Participants self-identified their ethnicity. We adapted outcome scales into Sylheti, Urdu and Punjabi; validation was by back translation using lay and expert panels.

### Study power and analysis

Without taking account of clustering, to detect a clinically important difference of 68% to 48% with 80% power for 5% significance requires 105 participants in each group.[37] To adjust for clustering and for variable cluster size [38] assuming the intracluster correlation coefficient (ICC) equals 0.05, the coefficient of variation of cluster size equals 0.65 and mean cluster size equals 3 (50% of cluster size in the ELECTRA trial [6] which recruited similar participants but across all ethnic groups) results in 123 participants in 41 clusters required in each group. Adding 2 extra clusters per group to allow for cluster drop out, and allowing for 15% loss to follow up of participants we require 304 individuals from 86 clusters. Given the small cluster size, we estimated that at least 5% of practices, and probably more, would not recruit any participants. Allowing further for some practices refusing to participate we approached all 107 practices in the two London boroughs of Newham and Tower Hamlets.

The data are summarised using descriptive statistics: means and standard deviations for continuous variables, frequencies and proportions for binary data and medians and Kaplan-Meier curves for the time-to-event data.

The time-to-event outcomes were analysed using Cox proportional hazards models, binary and count outcomes using generalised estimating equations (GEE), assuming an exchangeable correlation structure, and with link functions and families appropriate to outcomes, continuous outcomes using linear regression, in each case allowing for clustering of patients within practice. On modeling each binary outcomes using a GEE, the ICC was obtained directly from the working correlation matrix. In the case where this ICC was negative, as can arise in situations described by Eldridge, we present results from the model not adjusted for clustering.[39] *A priori*, we chose to adjust for the following baseline covariates in order to improve the precision of the treatment effect estimate (continuous outcomes) and to obtain a more accurate estimate of the treatment effect (binary and time-to-event outcomes): the outcome measured in the 12 months before randomisation, asthma severity assessed by medication step (BTS step 3, 4 or 5 vs. 1 or 2), the stratification and minimisation factors (Borough, numbers of GPs, practice asthma admission rate, percent practice list south Asian, numbers of south Asians).

We did not perform any multiple imputation. All analyses were performed using all available data following intention-to-treat principles.

In planned subgroup analyses, we tested whether treatment effects differed by ethnicity and whether child or adult. All analyses were carried out in Stata release 10.[40]

## Results

**Fig 2:**
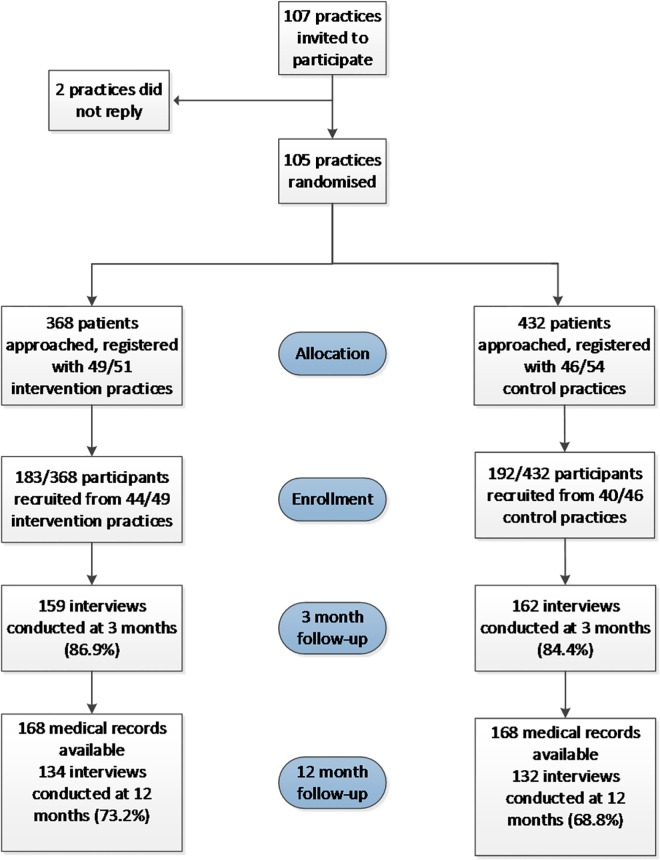
Flow of practices and patients through the study

Practices in the control and intervention groups were comparable for practice characteristics, and stratification and minimisation factors (Table 1). The CONSORT flow diagram (Fig 2) shows the flow of practices and participants through the study. Characteristics of participants are given in Table 2.

**Table 1:** Practice characteristics used in the minimisation, stratified by Borough

		Newham		Tower Hamlets	
		Number of practices		Number of practices	
		(N = 66)		(N = 39)	
Minimisation variable	Categories	Intervention	Control	Intervention	Control
Number of	1	20	19	6	7
GP partners	2	8	6	5	7
	3+	6	7	6	8
Asthma	low (≤1)	10	10	5	5
admission	medium (>1 & ≤1.5)	16	14	5	8
rate	high (>1.5)	8	8	7	9
Per cent South Asian patients registered with practice	≤15%	8	11	4	6
	>15% to ≤30%	14	12	9	9
	>30%	12	9	4	7
Number of	≤ 600	21	20	10	10
South Asians	>600 & ≤1200	11	10	4	7
registered	>1200	2	2	3	5

**Table 2:** Patient characteristics

		Intervention		Control	
N	(practices)		44		40
n	(patients)	183	100%	192	100%
Sex (M/F)	(%male)	98/85	54%	99/93	52%
Age	(mean & SD)	24.8 (21.6)		25.2 (20.9)	
Age ended education	n (mean & SD)	50	18.0 (4.3)	54	18.4 (5.0)
Adult/child	(%adult)	90/93	49%	99/93	52%
Smoker	Yes	6/90	7%	7/99	7%
Housing	council	58	32%	90	48%
	rented	27	15%	19	10%
	owner	98	53%	78	42%
Marital Status (adults only)	married	55	63%	65	68%
	single/widowed/divorced/separated	33	37%	31	32%
BTS step	3, 4 or 5 (%)	45	25%	40	21%
Generation in UK	1st	60	33%	68	35%
	2nd	76	41%	70	37%
	3^rd^ or 4^th^	47	26%	54	28%
Ethnic Origin	Bangladeshi	58	31%	94	49%
	Indian	13	7%	6	3%
	Pakistani	18	10%	10	5%
	Sri Lankan	16	9%	2	1%
	British Bangladeshi	23	13%	38	20%
	British Indian	22	12%	16	8%
	British Pakistani	29	15%	23	12%
	British Asian	3	2%	2	1%
	African Indian	1	1%	1	<1%
Fluent in English	Yes	137	75%	117	61%

Practices contributed a median of three participants to the study; 21 practices contributed none. Characteristics of participants are given in Table 2. The groups were reasonably comparable, for example similar proportions by gender and asthma severity, expressed as British Thoracic Society on medication step 3, 4 or 5 (severe), although the control group had more Bangladeshi patients than the intervention group (142 vs. 81), and the intervention group had more patients of Indian, Pakistani or Sri Lankan origin (98 vs. 57). The majority of patients were recruited at an A&E attendance (56%).

Fifty percent of participants were adults. Median age in intervention group: 14.8 years; in control group: 16.6 years. Overall median age was 15.4 years compared with a mean age of 25 years (SD = 21). Approximately 6% were smokers, around one third of adults were married and just under a quarter were at BTS Step 3 or above. A third were first generation immigrants in the UK and the majority were fluent in English, though this differed between intervention and control groups (75% vs. 61%).

### Intervention

Outpatient appointments were attended by 165/183 (90%) of intervention participants where they were reviewed by a PACE-trained asthma specialist nurse. Interpreters were used in 43 (23%) of these consultations (only 46 intervention patients rated themselves not fluent in English). All 165 intervention patients were followed up at least once (mainly by telephone) by the nurse to reinforce messages, with 148 and 55 having a second and third follow up consultation respectively. One hundred and fifty nine (87%) participants were recorded as agreeing short term goals for their asthma, and 158 (86%) had a treatment plan. One hundred and twelve (61%) participants were given a formal written self management plan. One hundred and forty two (78%) intervention patients had a review appointment made with their general practice by the asthma specialist nurse. One hundred and three (56%) agreed to attend a CDSMP group but only 44 of these (43%) did, with 19 patients attending three or more sessions of the six session course.

### Primary outcome: unscheduled asthma care (Table 3)

**Fig 3:**
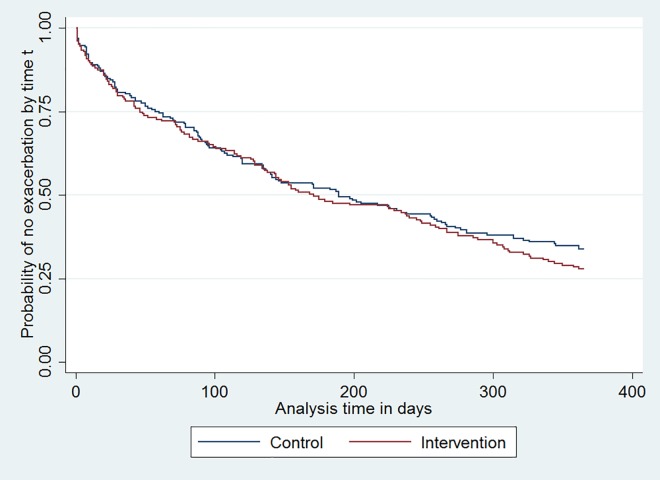
Kaplan-Meier estimate of probability of attending for first episode of unscheduled asthma care by days since intervention

Primary outcome data were available for 358/375 (95.5%) of study participants. The intervention had no effect on time to unscheduled care (median time to event 171 days in intervention patients, 189 in the controls, (Fig 3), adjusted hazard ratio (AHR) = 1.19, 95% CI 0.92 to 1.53, P = 0.185). The ICC from the adjusted model for the binary co-primary outcome was -0.028. The intervention was found to have had no effect on the percentage of participants without unscheduled care (adjusted odds ratio (AOR) = 0.71 (0.43 to 1.20), P = 0.202).

**Table 3:** Primary outcomes Median time to first unscheduled care: 171 days (Intervention), 189 days (control)

Primary outcomes	Effect estimate	95% CI	P-value
Time to first unscheduled care*^a^	AHR = 1.19	0.92 to 1.53	0.185
Proportion without unscheduled care*^b^	AOR = 0.71	0.43 to 1.20	0.202

*adjusted for patient’s baseline count of unscheduled care contacts, BTS Step (3, 4 or 5 vs. 1 or 2), Primary Care Trust, number of GP partners, practice asthma admission rate, per cent of practice list that is south Asian and number of south Asians on practice list.

^a^The standard errors are adjusted for clustering by practice

^b^Unclustered analysis due to negative ICC

AHR = adjusted hazard ratio, AOR = adjusted odds ratio.

### Secondary outcomes

#### Review of asthma in primary care (Table 4)

**Fig 4:**
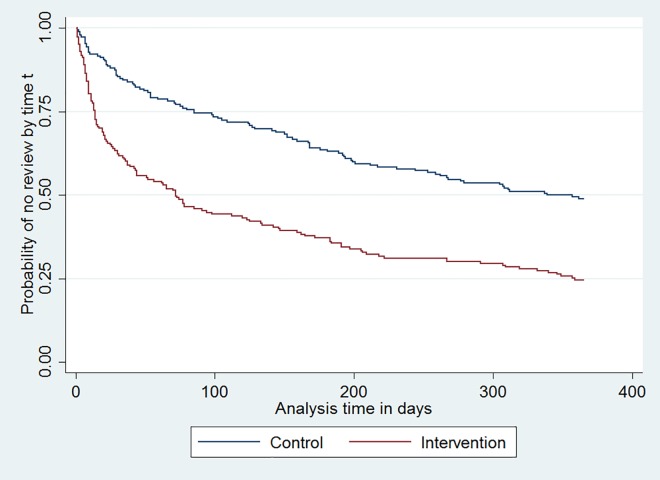
Kaplan-Meier estimate of probability of attending for first asthma review by days since intervention

The intervention significantly shortened the time to an asthma review consultation in primary care (AHR = 2.22 (1.67 to 2.95) P<0.001, Fig 4). The ICC from the adjusted model for asthma review within three months was -0.023. Therefore, fitting an unclustered model, the odds of being reviewed within three months increased almost four-fold for intervention patients compared to controls (AOR = 3.70 (2.29 to 5.96), P<0.001).

**Table 4:** Secondary outcomes from medical records. Median time to review 72 days (Intervention), 339 days (control)

Secondary outcomes	Effect estimate	95% CI	P-value
Time to first review in primary care*^a^	AHR = 2.22	1.67 to 2.95	< 0.001
Proportion having a review within 3 months*^b^	AOR = 3.70	2.29 to 5.96	< 0.001

*adjusted for patient’s baseline count of review contacts, BTS Step (3, 4 or 5 vs. 1 or 2), Primary Care Trust, number of GP partners, practice asthma admission rate, per cent of practice list that is south Asian and number of South Asians on practice list.

^a^Proportional hazards assumption not met so interaction between baseline count of outcome and logarithm of analysis-time also fitted

^b^Unclustered analysis due to negative ICC

AHR = adjusted hazard ratio, AOR = adjusted odds ratio.

#### Quality of life, symptoms, and self efficacy (Tables 5 and 6)

Three months after intervention, patients reported improvements in quality of life measured by the AQ20 scale (but not the EQ5D scale), symptoms (North of England Scale) and asthma self efficacy. Differences reaching statistical significance for quality of life and asthma self efficacy only (Table 5). These differences had waned and were no longer statistically significant by twelve months after intervention (Table 6).

**Table 5:** Secondary outcomes from interviews held at 3 months after intervention

		Intervention			Control						
		n	mean	SD	n	Mean	SD	Adjusted mean difference	95% CI for difference	P-value	Point estimate favours
EQ-5D (quality of life)	Baseline	68	0.78	0.30	72	0.76	0.25	0.04	-0.05 to 0.12	0.374	Intervention
	3 months	68	0.83	0.29	72	0.82	0.29				
AQ20 (quality of life)	Baseline	71	13.3	4.5	75	13.8	4.3	-2.56	-3.89 to -1.24	<0.001	Intervention
	3 months	71	10.8	5.9	75	13.3	4.7				
North of England (symptoms)	Baseline	158	13.4	5.4	162	13.7	5.2	-0.64	-1.55 to 0.28	0.169	Intervention
	3 months	158	9.5	4.9	162	10.4	4.8				
Asthma self-efficacy	Baseline	152	5.9	2.1	160	5.9	1.9	0.44	0.05 to 0.82	0.027	Intervention
	3 months	152	6.7	2.1	160	6.3	1.9				

In each model, the standard errors are adjusted for clustering by practice and for baseline value of outcome, BTS Step (3,4 or 5 vs. 1 or 2), number of GP partners, practice asthma admission rate, per cent of practice list that is south Asian and number of south Asians on practice list. SD is standard deviation

**Table 6:** Secondary outcomes from interviews held at 12 months after intervention

		Intervention			Control						
		n	Mean	SD	N	Mean	SD	Adjusted mean difference	95% CI for difference	P-value	Point estimate favours
EQ-5D(quality of life)	Baseline	54	0.74	0.32	56	0.76	0.24	-0.04	-0.12 to 0.04	0.357	control
	12 months	54	0.82	0.25	56	0.87	0.21				
AQ20(quality of life)	Baseline	55	13.6	4.6	56	13.9	3.7	-0.78	-2.58 to -1.02	0.391	Intervention
	12 months	55	11.5	5.9	56	12.3	5.5				
North of England (symptoms)	Baseline	133	13.3	5.4	131	13.5	5.1	-0.04	-1.16 to 1.09	0.949	Intervention
	12 months	133	9.9	5.0	131	10.1	4.2				
Asthma self-efficacy	Baseline	129	5.7	2.1	131	5.9	1.9	0.25	-0.13 to 0.63	0.188	Intervention
	12 months	129	6.4	1.8	131	6.3	1.6				

In each model, the standard errors are adjusted for clustering by practice and for baseline value of outcome, BTS Step (3,4 or 5 vs. 1 or 2), number of GP partners, practice asthma admission rate, per cent of practice list that is south Asian and number of south Asians on practice list. SD is standard deviation

#### Changes in prescribing

In the 12 month period prior to the intervention, intervention patients had, on average, 0.75 antibiotic prescriptions, control patients 0.68. These had increased at the primary endpoint to 0.95 and 1.04 respectively. However, there was no evidence of a difference between groups: adjusted incidence rate ratio (AIRR) 1.12, 95% CI (0.91 to 1.39). In the intervention group, the mean number of inhaled steroid prescriptions was 0.76 at baseline, 0.58 in the controls. At the primary endpoint this had increased to 1.16 and 0.98, with no evidence of a difference between the groups AIRR = 1.14, 95% CI (0.87 to 1.49)

No harms were detected.

### Subgroup analyses

We carried out pre-planned moderator analyses for ‘time to first review in primary care’—the only major significant treatment outcome. These analyses explored the effects of ethnicity, whether the patient was an adult or a child, whether or not the patient was fluent in English, and whether or not the patient was a first generation immigrant. We collapsed the variable ‘ethnicity’ into three categories: Bangladeshi, British Bangladeshi and ‘other South Asians’ (the reference category, essentially comprising Indian, Pakistani or Sri Lankan).

There was evidence that ethnicity moderated the intervention effect (P = 0.046). The hazard ratio (AHR = 1.55) was greatest (best outcome–i.e. earlier review in primary care) for ‘other South Asians’ in the intervention group, close to 1 (AHR = 1.11) for the Bangladeshis and below 1 for British Bangladeshis (AHR = 0.72) (poorer outcome). In the control group the AHRs were much lower than 1, indicating a greater time to first asthma review in primary care in the Bangladeshis (AHR = 0.46), and was lowest in the British Bangladeshis (AHR = 0.17).

There was strong evidence of effect moderation in children compared to adults (P = 0.0003). Adults (AHR = 1.44) in the intervention group had a better outcome (i.e. were reviewed earlier) than children, who did not appear to benefit from the intervention (AHR = 0.94). Children in the control group were the most likely to be reviewed later (AHR = 0.27).

There was strong evidence (P = 0.0004) that immigrant generation in the UK moderated the intervention effect: first generation immigrants in the intervention group had the best outcome (i.e. were reviewed earlier, AHR = 5.55), followed by first generation immigrants in the control group (AHR = 4.56) and then higher generation immigrants in the intervention group (AHR = 3.12). There was weak evidence (P = 0.083) that fluency in English moderated the effect of the intervention with review sooner in patients not fluent in English (AHR = 1.61).

## Discussion

### Summary

This intervention, directed at clinicians and their south Asian patients, failed to reduce unscheduled care for asthma. Other, more effective, interventions are needed to reduce unscheduled care in this population. Our intervention did, however, improve patients’ quality of life and self-efficacy (confidence to control asthma) and improved follow up in primary care, shortening time to review and increasing the proportion followed up. To our knowledge this is the first intervention to target specifically South Asians with asthma and their clinicians.

Our study was unusual in that it targeted both patients and their clinicians. Specialist nurses successfully implemented the main elements of the culturally adapted PACE program, with almost nine in every ten patients agreeing both short term goals and a treatment plan for their asthma care, and more than six in every ten receiving a written self management plan. These, combined with early review in primary care by PACE-trained primary care teams probably account for the improved quality of life and confidence to control asthma found in patients in the intervention group. These changes were insufficient to influence attendance for unscheduled care. We found no significant improvement in asthma symptoms, suggesting that asthma control and adherence were unchanged. Low uptake of CDSMP sessions by intervention patients may have reduced reinforcement of self-management behaviours such as goal-setting and action planning. Alternative explanations include a failure to affect behaviours that trigger consultations during asthma exacerbations, or a failure to address biomedical factors that might affect asthma control. Vitamin D deficiency is common in south Asian groups in east London[41] (90% insufficient, 50% deficient), which may contribute to poor asthma control and increased risk of exacerbation,[42, 43] potentially rendering behavioural interventions less effective. Further work should address these factors.

### Strengths and weaknesses

The study used a large, robust cluster randomised design. Successful recruitment of almost all general practices across two London boroughs, and recruitment of a large proportion of eligible patients increase the generalisability of the findings. Patients recruited had significant asthma with over one third having a hospital admission and over three quarters having at least one ER visit in the previous year, and so had potential to gain from the intervention. Use of medical records meant that data was more than 95% complete for the primary outcome, adding strength to our conclusions

The main weakness was the lower than expected number of patients attending the CDSMP sessions.

### Comparison with other data

To our knowledge, only one other randomised trial has targeted south Asians with asthma.[15] In that UK-based study, Moudgil found that a 40 minute self-management education session for 689 white and south Asians adults and children with asthma, reinforced by telephone after four and eight months, did not reduce unscheduled care in south Asians but did improve quality of life.[15]. Other studies, all set in the USA and mostly small in size, have targeted African Americans or Hispanics using group interventions,[17, 19] individual interventions,[12–14] and family-based interventions for children,[11, 18] with inconsistent results. All involved multiple education sessions, often combining in-patient [12, 16] or home-based education,[11, 18] some with telephone re-enforcement. Some adapted existing interventions,[13, 18] while others were developed *de novo*.[11, 19] Explicit basing of interventions on educational theory was rare. No study targeted clinicians as well as patients, although this approach has been effective in school-based asthma interventions.[44] No one of these strategies appears to be consistently effective, although family-based interventions[11, 18] and involvement of social/community workers shows promise.[12, 18].

### Clinical and research implications

Improving asthma morbidity for minority populations remains a significant challenge, particularly for south Asian groups. These interventions appeared less effective in the UK than in the USA. Reasons are unclear but there are major differences in heath care systems in the two countries. Caution should be exercised when assuming that interventions are transferrable across health systems and cultures.

It is likely that, if interventions are to impact on health care use, they need to be both intensive and delivered in a culturally-specific way. Further work should clarify the relative merits of *de novo* development *versus* adaptation of pre-existing interventions, and explore further the impact of family-based interventions for children, and involving community workers. Cost-effectiveness analyses should form a part of all evaluations, given the intensity of interventions required. The role and / or importance of educational theory in the development and delivery of interventions is not clear from existing studies.

In summary, morbidity remains high among ethnic minority groups with asthma, yet few interventions have been tested and fewer found to be effective. The development of cost-effective interventions to reduce morbidity in minority populations with asthma should remain a high priority.

## Supporting Information

S1 File Consort checklist (DOCX)

S2 File Trial protocol (DOCX)
