# Supplementary material for: Effect of an Education Programme for South Asians with Asthma and Their Clinicians: A Cluster Randomised Controlled Trial (OEDIPUS)
Source: PLoS One. 2016 Dec 28;11(12):e0158783. doi: 10.1371/journal.pone.0158783 (PMC5193334; doi:10.1371/journal.pone.0158783)
Supplement: S2 File — (DOCX) [file pone.0158783.s002.docx]

**Education programme for south Asians with asthma and their primary and secondary care clinicians: a cluster randomised controlled trial (ŒDIPUS)**

**Study Protocol**

**a) Original hypothesis:** an intervention comprising education for south Asians with asthma and their primary and secondary care clinicians will reduce unscheduled care.

**b) Background:** Health inequalities between ethnic minority and majority groups exist for all chronic diseases and are a government priority for action.^1^ For asthma, poorer outcomes for people from minority groups are a universal finding*.*^2-5^ In New York, a black person with asthma is up to nine times more likely to die from asthma than people from other ethnic groups.^2^ In the UK, hospital admission rates for all minority groups exceed those of whites (roughly double for south Asians).^5-7^ Improving outcomes for minority groups is one of the most difficult challenges for health services. No randomised trials have reduced emergency asthma care for ethnic minority groups, although one improved routine care,^8^ and other small studies have reduced emergency care in mixed white and minority populations.^9;10^ Improving outcomes in minority groups is vital: if health care interventions benefit white majority groups preferentially, the net effect is perversely to widen health inequalities.

Over the past five years we have developed a programme of qualitative and quantitative work^11-18^ that:

supports suggestions^19^ that white populations benefit preferentially from asthma education,^15^

identifies key cultural and structural barriers to improvements in asthma control in south Asians,^11^ ^14;16^

provides the first randomised evidence for benefit of lay-led educational interventions in a south Asian population.^18^

Barriers to improving asthma control in south Asians include:

**Poor access to routine care^20^**

**Poor follow up** after A&E attendance or admission; only 20% are reviewed within 3 months^15^

**Language barriers in consultations^21^**

**Poor understanding of asthma and medication, and lack of shared explanatory models between clinician and patient.^16^**  The explanatory models of asthma held by people of Bangladeshi origin living in the UK are less likely than those of white people to contain biomedical concepts of prevention and structural notions of airways obstruction, making advice about medication less effective.

**Cultural attitudes to, and difficulties learning self-management.** The concept of self-management has less currency (particularly in Islamic cultures) where behaviour may be more related to family or religious identity than the self.^11;16;22^ In addition, difficulties gaining adequate information to guide self-care contribute to low self-efficacy (itself a Western concept) and passive approaches to illness.^14^

**Less frequent shared decision making** in consultations reducing adherence to medication and behavioural advice,^16^ and differences in concepts of patient-doctor partnership amongst whites and south Asians.^12^

Factors 1 and 2 reduce access to care; factors 3, 4, 5 and 6 reduce the effectiveness of consultations.

We propose to evaluate an intervention developed specifically to addresses these barriers. Our trial uses a similar design to our successful ELECTRA study, which tested a specialist nurse intervention:^15^

The target group is south Asian people aged 3 and above attending hospital for acute asthma. The intervention comprises evidence-based components:^23^

**Education of specialist nurse and primary care clinicians to improve the effectiveness of follow-up consultations after hospital attendance a) by specialist nurses and b) in primary care**. This will comprise an adaptation of Clark’s self-regulation intervention.^24^ This promotes shared decision-making and partnerships with patients. It was impressively successful in improving asthma outcomes by paediatricians in the USA.^24^

**Education of patients using lay-led small group ‘expert-patient’ sessions** to improve understanding of asthma, and promote confidence and the concept of self-management, using an asthma-specific adaptation of Lorig’s Chronic Disease Self Management Programme (CDSMP).^25^ Our trial of a Bengali adaptation of the CDSMP (Expert Patient Programme) is the first RCT to demonstrate improved outcomes (self-efficacy, coping, reduced depression) of an educational intervention in south Asians.^18^

**Improved follow-up in primary care after hospital attendance**, using a simple method of appointment booking.^26^

The setting is Newham and Tower Hamlets, boroughs/PCTs with the UK’s 1^st^ and 3rd highest ethnic minority populations. Newham has a mixed population (12% Indian;9% Bangladeshi); Tower Hamlets largely Bangladeshi (33%[largest Sylheti population outside Bangladesh]). Addressing respiratory inequalities is a priority for both boroughs: this project forms part of Tower Hamlets’ Health Improvement Programme and is welcomed by Newham’s Asthma Scrutiny Programme.

**c) Design**

Pragmatic cluster randomised controlled trial, randomising general practices.

***Participants****:*

*Practices –* We will invite all 94 Tower Hamlets and Newham practices to take part. These have mean 21% (range:0.3-85%) south Asian patients; (82 are above UK population mean 3.6%). We will use recruitment methods proven successful in our recent studies (90-100% of practices)^15;27-29^ – peer researcher GPs writing to practices giving study information, with follow-up telephone call requesting participation.

*Patients -* We will invite prospectively all south Asians (Bangladeshi, Pakistani, Indian, Sri Lankan, identified by name and self-categorisation) aged 3 years and above attending the Royal London and Newham General hospitals with physician-diagnosed acute asthma (admissions, A&E attendances or out-patient visits) or GP out-of-hours/NHS walk-in centres (excluded if co-diagnosis of COPD, defined as lack of bronchial reversibility). Research assistants will invite them to attend specialist nurse-run clinics at the London and Newham hospitals, to obtain informed consent, demography and FEV1. This method was successful in the ELECTRA study. 2001 Census suggests approx 70% will be Bangladeshi, with remainder from other groups.

***Cluster randomisation of practices***

We will stratify practices by partnership size, training status, practice nurse employment and previous asthma admission rate, and randomise them to intervention or control groups using a computer minimization programme.^15^

***Intervention***:

1. *Education of specialist nurse and primary care clinicians – the PACE PROGRAMME*. We have adapted Clarke’s self regulation intervention making it appropriate to promote culturally competent consulting with south Asians. This already emphasizes the notion of clinician-patient partnership. Our adaptation of the video and coursework comprises increased emphasis on: a) the nature and causes of airways obstruction; b) the roles of, and rationale for medication; c) taking personal control of asthma; d) effective use of bilingual advocates.^21^ We will invite the trial specialist nurse and GPs/practice nurses from the intervention group to attend two three hour education sessions, in groups of 10-15. Participants receive a video and course material for homework between sessions. Educating the specialist nurse and primary care clinicians together will improve mutual understanding of roles and communication.
2. *Consultations by asthma specialist nurse at hospital nurse-run clinics.* The self-regulation education will promote a more effective consultation style, specifically promoting: non-verbal attentiveness and encouragement, interactive conversation and praise for things done well, exploration of concerns with appropriate reassurance, agreement on short and long term goals, and criteria for making management decisions. Where possible, a written treatment plan will be provided (Sylheti has no written form; some Bangladeshis read standard Bengali). Contamination will be avoided by having a single specialist nurse for intervention participants covering Tower Hamlets and Newham, with existing specialist nurses providing usual care for control patients. Bilingual health advocates will be available for intervention and control patients. In addition the intervention specialist nurse will arrange:
   1. *Follow-up appointment in primary care.* The specialist nurse will telephone the participant’s general practice to make them a convenient appointment for follow-up by the practice nurse or GP, and will send detailed hospital/A&E discharge planning information^26;30^ including education given and medication changes. The importance of primary care altering their medication records will be emphasized.
   2. *Appointment for lay-led self-management education.* The specialist nurse will make appointments for participants to attend a six week lay-led self-management education programme.
3. *Lay*-*led self-management education – the CHRONIC DISEASE SELF MANAGEMENT PROGRAMME,* comprises our *asthma-specific adaptation* of the Lorig Chronic Disease Self Management Programme - six weekly three hour small group education sessions to promote understanding of the mechanisms and treatment of asthma, and the concept of self-management of illness and specific self management skills. The asthma-specific component is based on lessons from our developmental work (including videotapes of 36 asthma consultations with interviews with both participants,^16^ and interviews and focus groups comprising 36 Bangladeshis attending lay-led self-management courses^11^), and is developed with Rowshan Khanem, experienced and accredited Expert Patient lay tutor and asthma-sufferer (who led our Bangladeshi Expert Patient sessions), and with other local Bangladeshis. The sessions will be led by pairs of trained and accredited lay tutors and incorporate simple visual aids to convey structural concepts of airways obstruction and reversibility with reliever and preventer medication. Educational video and written material being developed by the NAC will supplement this. Sessions will take place at convenient sites in or near the London and Newham hospitals. The tutor will encourage attendance (sessions confirmed in writing, telephone reminders, £10 voucher for attendance at sessions)*.* Tower Hamlets sessions will comprise almost exclusively Bengalis and will use the Sylheti dialect adaptation of the programme. Newham sessions will comprise a range of south Asians groups and will use English or Hindi as the main language (most Newham south Asians have a good grasp of English). We will run separate programmes for men, women, and for parents of children with asthma (with children aged 13 or above able to attend). These six week programmes will run back to back for each group during the intervention phase of the study. Thus at any one time there will be in progress Sylheti male, female and child/parent sessions in Tower Hamlets and Hindi male, female and child/parent sessions in Newham. Courses will run over an 18 month period, we expect to run 60 courses in all.
4. *Follow-up consultations in primary care*. The self-regulation education for clinicians should improve the effectiveness of consultations of practice nurses and GPs. Specific tasks are: reinforcing specialist nurse advice; implementing changed medication regimens; preventing further attacks.

***Control*** comprises usual specialist nurse care with usual discharge flimsy/letter.

***Outcomes***

*The primary outcome:* unscheduled care as *time to first unscheduled contact with an asthma exacerbation* and *proportion of participants without unscheduled care,* extracted from GP and hospital records (to ensure complete capture) by blinded researchers, with validation of random sample by another blinded researcher. We will extract data for the year before and year after intervention. The former is a vital covariate, being the most important predictor of unscheduled care.^15^ Unscheduled care is defined as emergency admission, A&E attendance, GP out-of-hours or walk-in centre asthma consultation, or GP consultation comprising presentation with acute asthma-related symptoms, with increase in asthma medication or antibiotic prescription.^15^

*Secondary outcomes*:

1. asthma-specific and generic HRQoL, using AQ20,^31^ North of England,^32^ and EQ5D^33^ scales assessed face-to-face at baseline and by telephone at 2 and 12 months. We have validated Sylheti versions of these and will develop Urdu and Punjabi versions.^17^
2. Asthma reviews (time to first/proportion with: primary care review <3 months)
3. Costs and prescribing assessed from patient records and interviews.

(Note: PEF/symptom diaries are not feasible)

*Study power:* The study is powered to detect a clinically important 20% reduction in the proportion of participants with unscheduled care (68% to 48%), with 80% power, 5% significance. With intracluster correlation co-efficient of 0.05, we require 300 participants. ELECTRA data shows this is feasible: baseline unscheduled care in ELECTRA was 68%; prospective recruitment of south Asians extrapolates in two hospital centres to 300 over 12 months; follow-up rate for primary outcome - 98%; average number of participants per practice - six.

***Statistical analysis***

We will analyse blind, using generalised estimating equations to allow for clustering by practice, with appropriate equations for different outcomes (binary/time-to-event/number of events), and exploratory hypothesis-generating analyses by ethnic sub-group. Outcomes expressed as Kaplan-Meier plots and hazard ratios.

***Health economics***

We will assess cost-effectiveness in terms of two questions:

1. What are the societal costs and benefits of implementing the programme?

2. What is the cost-effectiveness of the programme over two years?

Costs will be obtained by recording units of resources used, and applying tariffs to each. Important units are GP/nurse consultations, prescriptions, over-the-counter drugs, hospital/A&E/outpatients visits, and bed-days.

*Participants’ costs* will be obtained from the patient interview and will include time seeking advice, travel, home-care and out-of-pocket expenses on additional drugs. Wage rates are an inaccurate measure of opportunity cost. We will apply tarifs based on the views of a subset of participants using techniques such as contingent valuation or conjoint analysis. **Primary outcomes** are unscheduled care prevented for cost-effectiveness, and EQ5D for the cost-utility analysis, calculated using incremental cost-effectiveness ratios.

*Note: A qualitative evaluation* will be funded separately to explore the relative importance/effectiveness of components of the intervention and validity of findings.^23^

**Difficulties we expect**

This is a complex trial covering two inner-city boroughs.

1. *recruiting practices* – practice recruitment is increased if the trial team includes local lead GPs for respiratory care and for small practices (we achieved 98% recruitment in our TB screening trial this way^29^) with payments to acknowledge time involved. We will offer control practices a £200 participation fee and intervention GPs £200 for attending both educational sessions.
2. *recruiting patients -*  Support of A&E departments is vital – lead clinicians for both have given their support. We have demonstrated feasibility of recruiting *via* A&E in the ELECTRA study. We will aim to recruit research officers fluent in south Asian languages.
3. *Follow-up –* We achieved 98% follow-up for the primary outcome in ELECTRA in a mobile population. We expect similar rates in this study.
4. *attendance at lay-led education –* Participants come from a deprived area, where racial harassment is common. We achieved adequate attendance in our trial of lay-led education using phone and written reminders, incentives and travel costs. We will obtain vouchers of £10 per participant (£1500) from local businesses.

**d) Milestones** -2 0 2 4 6 8 10 12 14 16 18 20 22 24 26 28

Practice recruitment xxx

Practice randomisation xxx

Clinician education xx

Setting-up patient recruitment xx

Patient recruitment (m3-17) xxxxxxxxxxxxxxx

Patient intervention (m3-21) xxxxxxxxxxxxxxxxxxxx

2 month interviews xxxxxxxxxxxxxxxxxxx

12 month interviews(m15-27) xxxxxxxxxxxxxxxxxxxx

Data extraction xxxxxxxxxxxxxxxxxx

Analysis(m27-28) xxx

Writing xxx

Collaborators comprise:

- ***Education****: Noreen Clarke*, Professor of Public Health, Michigan University, providing expert advice on adaptation of educational intervention and implementation. *Vivien Cook,* Lead in Education and Training, Queen Mary.
- ***Trial coordinator****: Gill Foster,* respiratory research nurse. Gill coordinated ELECTRA.
- ***Secondary care:*** *Lead respiratory physicians, paediatricians and A&E consultants* in both hospitals: Professor Barnes, Drs Packe, McKenzie, Gopinathan, Oliver, Hobart, Lessof and Coats will contribute to successful recruitment.
- ***Tower Hamlets and Newham PCTs:*** *Directors of Public Health:* Drs Leahy and Hayward; *Lead respiratory GPs*: Drs Livingstone, Hull and Trathen.
- ***Consumer:*** *Social Action for Health:* east London community group who established the Bengali lay-led self-management programme will contribute to the steering group, providing advice on lay-led education. Rowshan Khanem – accredited chronic disease self-management programme tutor has helped design and will deliver/oversee the lay-led education.
- ***Academic:*** *Queen Mary’s School of Medicine, Bart’s and the London, Centre for General Practice and Primary Care:* Professors Griffiths and Feder, Drs Eldridge (statistician), Taylor (Public Health), and Spencer (economist); *Academic Respiratory Care:* Professor Barnes; *Paediatrics:* Dr MacKenzie.

Members of the above will form a steering group.

1. Acheson, D. Inequalities in health. 1998. London, HMSO.

2. Tanne JH. Asthma "crisis" for black Americans. *BMJ* 2001;**323**:302.

3. Ehrlich RI,.Bourne DE. Asthma deaths among coloured and white South Africans: 1962 to 1988. *Respir.Med.* 1994;**88**:195-202.

4. Ellison-Loschmann L, Cheng S, Pearce N. Time trends and seasonal patterns of asthma deaths and hospitalisations among Maori and non-Maori. *N.Z.Med.J* 2002;**115**:6-9.

5. Gilthorpe MS, Lay-Yee R, Wilson RC, Walters S, Griffiths RK, Bedi R. Variations in hospitalization rates for asthma among black and minority ethnic communities. *Respir.Med.* 1998;**92**:642-8.

6. Ormerod LP. Adult Asian acute asthma admissions reassessed: Blackburn 1991-1992. *Respir.Med.* 1995;**89**:415-7.

7. Myers P,.Ormerod LP. Increased asthma admission rates in Asian patients: Blackburn 1987. *Respir.Med.* 1992;**86**:297-300.

8. Evans D, Mellins R, Lobach K, Ramos-Bonoan C, Pinkett-Heller M, Wiesemann S *et al*. Improving care for minority children with asthma: professional education in public health clinics.[comment]. *Pediatrics* 1997;**99**:157-64.

9. George MR, O'Dowd LC, Martin I, Lindell KO, Whitney F, Jones M *et al*. A comprehensive educational program improves clinical outcome measures in inner-city patients with asthma.  *Arch.Intern.Med* 1999;**159**:1710-6.

10. Castro M, Zimmermann NA, Crocker S, Bradley J, Leven C, Schechtman KB. Asthma intervention program prevents readmissions in high healthcare utilizers. *Am.J Respir.Crit Care Med* 2003;**168**:1095-9.

11. Motlib, J., Azad, A., Khanem, R., Ramsay, J., Tahir, R., Eldridge, S., Garrett, M., Barlow, J., Feder, G., and Griffiths, C. Impact of a lay-led chronic disease self-management programme for Bangladeshi people: quantitative and qualitative evaluation. Society for Academic Primary Care. 2003.

12. Car, J., Freeman, G. K., Griffiths, C., Sheikh, Aziz, Rhodes, T., Ashcroft, R. E., and Lais, M. Exploring patient-doctor partnership in white European and south Asian adults with asthma: qualitative interview study. University of Wales Swansea. The 2nd International Shared Decision Making Conference: "New Conversations with Patients"; abstract 93, 2003.

13. Griffiths, C., Kelly, M., and Jones, I. R. Asthma and psychosocial adversity: a pilot study, in Lower Clapton Group Practice Primary Care Research Team Annual Report. 2002. London, Lower Clapton Group Practice.

14. Griffiths C, Kaur G, Gantley M, Feder G, Hillier S, Goddard J *et al*. Influences on hospital admission for asthma in south Asian and white adults: qualitative interview study. *BMJ* 2001;**323**:962.

15. Griffiths, C., Foster, G., Barnes, N., Eldridge, S., Tate, H., Begum, S., Wiggens, M., Dawson, C., Livingstone, A., Coats, T., Chambers, M., and Feder, G. A specialist nurse intervention to reduce unscheduled care for asthma in a deprived multiethnic area: the East London randomised controlled trial for high-risk asthma (ELECTRA). BMJ . 2003.

16. Foster, G. and Griffiths, C. Ethnic differences in consultations for asthma: a qualitative and quantitative study. Society for Academic Primary Care, accepted. 2003.

17. Griffiths C, Ahmed S, Ahmed S, Nandy S, Abrams C, Meadows KA *et al*. Using health-related quality of life measures in minority ethnic groups: an approach to translating measures into Bengali (Sylheti). *European Journal of General Practice* 2000;**6**:130-4.

18. Griffiths, C., Ramsay, J., Azad, A., Motlib, J., Eldridge, S., and Feder, G. Expert Bangladeshi patients? A randomised trial of a lay-led self management programme for Bangladeshis with respiratory and cardiovascular disease. European Respiratory Journal 22: S45, 409s. 2003.

19. Moudgil H, Marshall T, Honeybourne D. Asthma education and quality of life in the community: a randomised controlled study to evaluate the impact on white European and Indian subcontinent ethnic groups from socioeconomically deprived areas in Birmingham, UK.[comment]. *Thorax* 2000;**55**:177-83.

20. Campbell JL, Ramsay J, Green J. Age, gender, socioeconomic, and ethnic differences in patients' assessments of primary health care. *Qual.Health Care* 2001;**10**:90-5.

21. Jones D, Gill P. Interpreting and translation. In Kai J, ed. *Ethnicity, health and primary care*, Oxford: Oxford University Press, 2003.

22. Sheikh A, Gatrad AR. Caring for Muslim Patients. Abingdon: Radcliffe Medical Press, 2000.

23. Campbell M, Fitzpatrick R, Haines A, Kinmonth AL, Sandercock P, Spiegelhalter D *et al*. Framework for design and evaluation of complex interventions to improve health. *BMJ* 2000;**321**:694-6.

24. Clark NM, Gong M, Schork MA, Evans D, Roloff D, Hurwitz M *et al*. Impact of education for physicians on patient outcomes. *Pediatrics* 1998;**101**:831-6.

25. Lorig, Kate, Sobel, D., and Stewart, A. Evidence suggesting that a chronic disease self-management program can improve health status while reducing hospitalization: a randomized trial. Medical Care 37(1), 5-14. 1999.

26. Baren JM, Shofer FS, Ivey B, Reinhard S, DeGeus J, Stahmer SA *et al*. A randomized, controlled trial of a simple emergency department intervention to improve the rate of primary care follow-up for patients with acute asthma exacerbations. *Ann.Emerg.Med* 2001;**38**:115-22.

27. Feder G, Griffiths C, Highton C, Eldridge S, Spence M, Southgate L. Do clinical guidelines introduced with practice based education improve care of asthmatic and diabetic patients? A randomised controlled trial in general practices in east London. *BMJ* 1995;**311**:1473-8.

28. Feder G, Griffiths C, Eldridge S, Spence M. Effect of postal prompts to patients and general practitioners on the quality of primary care after a coronary event (POST): randomised controlled trial. *BMJ* 1999;**318**:1522.

29. Sturdy, P., Brewin, P., Griffiths, C., Bothamley, G., Feder, G., McDonald, M., Tibrewal, S., Graffy, J., Eldridge, S., Sloan, D., Ramsay, J., and Zumla, A. Is screening for tuberculosis effective? A cluster randomised trial in east London general practices? European Respiratory Journal 22: S45, 200s. 2003.

30. Osman LM, Calder C, Godden DJ, Friend JA, McKenzie L, Legge JS *et al*. A randomised trial of self-management planning for adult patients admitted to hospital with acute asthma. *Thorax* 2002;**57**:869-74.

31. Barley EA, Quirk FH, Jones PW. Asthma health status measurement in clinical practice: validity of a new short and simple instrument. *Respir.Med.* 1998;**92**:1207-14.

32. Steen N, Hutchinson A, McColl E, Eccles MP, Hewison J, Meadows KA *et al*. Development of a symptom based outcome measure for asthma. *BMJ* 1994;**309**:1065-8.

33. EuroQol Group. EuroQol: a new facility for measurement of health related quality of life. *Health Policy* 1990;**16**:199-208.
